# The Effect of Brain Anodal and Cathodal Transcranial Direct Current Stimulation on Psychological Refractory Period at Different Stimulus-Onset Asynchrony in Non-Fatigue and Mental Fatigue Conditions

**DOI:** 10.3390/brainsci14050477

**Published:** 2024-05-08

**Authors:** Somayeh Hafezi, Mohammadreza Doustan, Esmaeel Saemi

**Affiliations:** Department of Motor Behavior and Sport Psychology, Faculty of Sport Sciences, Shahid Chamran University of Ahvaz, Ahvaz 6135783151, Iran; s.hafezii87@gmail.com (S.H.); e.saemi@scu.ac.ir (E.S.)

**Keywords:** posterior lateral prefrontal cortex, response selection, multitasking, bottleneck, dual task, resource sharing

## Abstract

The psychological refractory period (PRP) effect occurs when two stimuli that require separate responses are presented sequentially, particularly with a short and variable time interval between them. Fatigue is a suboptimal psycho-physiological state that leads to changes in strategies. In recent years, numerous studies have investigated the effects of transcranial direct current stimulation (tDCS) on motor control. The present study aimed to investigate the effects of two tDCS methods, anodal and cathodal, on PRP in ten different conditions of stimulus-onset asynchronies (SOAs) under non-fatigue and mental fatigue conditions. The participants involved 39 male university students aged 19 to 25 years. In the pre-test, they were assessed using the PRP measurement tool under both non-fatigue and mental fatigue conditions. The mental fatigue was induced by a 30-min Stroop task. The test consisted of two stimuli with different SOAs (50, 75, 100, 150, 300, 400, 600, 900, 1200, and 1500 ms). The first was a visual stimulus with three choices (letters A, B, and C). After a random SOA, the second stimulus, a visual stimulus with three choices (colors red, yellow, and blue), was presented. Subsequently, participants were randomly assigned to the anodal, cathodal, and sham stimulation groups and underwent four consecutive sessions of tDCS stimulation. In the anodal and cathodal stimulation groups, 20 min of tDCS stimulation were applied to the PLPFC area in each session, while in the sham group, the stimulation was artificially applied. All participants were assessed using the same measurement tools as in the pre-test phase, in a post-test phase one day after the last stimulation session, and in a follow-up phase four days after that. Inferential statistics include mixed ANOVA, one-way ANOVA, independent, and dependent t-tests. The findings indicated that the response time to the second stimulus was longer at lower SOAs. However, there was no significant difference between the groups in this regard. Additionally, there was no significant difference in response time to the second stimulus between the fatigue and non-fatigue conditions, or between the groups. Therefore, tDCS had no significant effect. There was a significant difference between mental fatigue and non-fatigue conditions in the psychological refractory period. Moreover, at lower SOAs, the PRP was longer than at higher SOAs. In conditions of fatigue, the active stimulation groups (anodal and cathodal) performed better than the sham stimulation group at higher SOAs. Considering the difference in response to both stimuli at different SOAs, some central aspects of the response can be simultaneously parallel. Fatigue also affects parallel processing. This study supports the response integration phenomenon in PRP, which predicts that there will be an increase in response time to the first stimulus as the interval between the presentation of the two stimuli increases. This finding contradicts the bottleneck model. In this study, the effectiveness of cathodal and anodal tDCS on response time to the second stimulus and PRP was found to be very small.

## 1. Introduction

In most sports fields involving open skills, such as rugby, football, basketball, handball, and many others, players must be able to make quick and accurate decisions in a complex and variable environment [1]. One strategy that competitive sports players often use to mislead and diminish their opponents’ performance is to deceive with a feint before executing the actual move [2]. One example of this deceptive maneuver is the head feint in basketball, where the player appears to be looking to the left but passes to the right at the same time, thereby thwarting the opponent’s effective defensive actions [3]. As a result, the defensive player is unable to complete a corrective response in time [4]. When the attacking player fakes, the defending player’s response to the second move can be delayed longer than when it is presented alone, giving the attacker a greater time advantage [5]. This delay creates a psychological refractory period (PRP), which is influenced by the onset asynchrony of stimuli [6]. The inability to overcome this distance and motor weakness can increase decision-making time [5], resulting in a delayed response to the second stimulus [7]. 

The effect of the PRP shows that the response time to the second stimulus increases as the time interval between the two stimuli decreases. Several models have been proposed to explain this effect, with the Response Selection Bottleneck (RSB) model [8,9] being the most dominant. Task processing is typically divided into three stages: perceptual processing, response selection, and response execution. Answer selection for two tasks cannot be performed simultaneously [10]. The effect of stimulus-onset asynchrony (SOA) on dual-task performance can be explained by the assumption that only the central processing steps of the two tasks [i.e., the step in which the appropriate response must be selected] can occur sequentially, while stimulus identification and response execution in a single task are considered in parallel with each stage of another task. This central bottleneck is conceptualized as an all-or-nothing phenomenon. The main reason for the prioritization of the first task over the second task is that stimulus identification is typically completed first in the first task, enabling it to reach the central stage [response selection] earlier [11]. This model predicts that there will be no change in response time to the first task if the second task is presented, as tasks are identified using the "in order out" method [12]. However, recent studies have challenged the assumption of purely serial central processing of response-related task features at different levels [13,14].

Hommel (1998) proposed the existence of partially parallel central processing across tasks [15]. His research shows that the choice of answer for the first task is influenced by the characteristics of the answer for the second task, and response activation in the second task can interfere with response-related processing in the first task [16]. According to this hypothesis, if the stimulus-response type is the same in two tasks, the presence of the second stimulus-response causes a delay in the response time of the first stimulus. This phenomenon is known as the backward compatibility effect (BCE) [15,17]. This suggests that at least some aspects of response-related central processing, such as response activation, can occur in parallel [17].

The central capacity-sharing (CCS) model [18,19] is similar to the central bottleneck model, but it divides the task into three processing stages, except that all stages can be performed in parallel with any other stage of a secondary task. This model assumes that limited cognitive capacity is continuously shared and differentially allocated between tasks during dual-tasking. A significant portion of the capacity is initially allocated to selecting the answer for the first task. This allocation is later [gradually or in stages] to complete the selection of the answer for the second task. Thus, instead of an inflexible all-or-none allocation mechanism based solely on the order of subtasks, this model allows for a flexible and graded sharing of capacity that can be adjusted to meet specific task needs or instructions [11]. 

Dux et al. (2006) showed delayed activity in the prefrontal cortex (PFC) in the PRP pattern, indicating that the frontal plays a crucial role in the central information processing bottleneck [20]. Previous studies using functional magnetic resonance imaging (fMRI) have localized this pathway in the left posterior lateral prefrontal cortex (PLPFC). The correlative evidence from these studies suggests that the PLPFC, particularly the inferior frontal junction (IFJ), plays a crucial role in response selection [20,21,22,23]. The IFJ area is believed to play a crucial role in cognitive control, decision-making, and selection, independent of the input method of task-related information [24,25]. Filmer et al. (2013a) demonstrated that the left PLPFC is a crucial neural site in the central bottleneck that restricts an individual’s capacity to make two simple decisions simultaneously. The left PLPFC is critical for higher levels of cognitive control and is organized into different functional sub-areas (Brodmann’s areas 9/46, 6/8/9, and 44/45) [26]. The left PLPFC is generally used specifically when processing demand increases [27]. Non-invasive neuromodulation techniques, such as transcranial direct current stimulation (tDCS), can provide a promising method to change the nervous, cognitive, and behavioral system functions [28]. tDCS is a well-known technique for modulating the excitability of the cerebral cortex. It has been widely used in neuroscience research due to its potential effects on the plasticity of the cerebral cortex, as well as its safety profile, ease of operation, affordability, and portability [29,30,31]. The application of this technique involves passing a low-intensity direct current (usually 0.2 to 2 mA) between pairs of electrodes placed on the scalp. This process creates an electric field in the underlying cortical tissue. The currents applied during tDCS modulate the resting membrane potential (between the membranes) of cortical neurons [32,33]. Whether neurons are depolarized or hyperpolarized depends on the direction of the electrical current relative to the neuronal orientation [34]. 

In both traditional and transcranial magnetic stimulation, one electrode is identified as the active electrode [target], and the other is identified as the return electrode (reference) [35]. By connecting the anodal (positive) and cathodal (negative) electrodes to specific areas on the surface of the skull, a weak direct current is established between the electrodes, resulting in the stimulation of subcortical neurons [36,37]. It is generally assumed that the anodal current depolarizes neurons, increasing the likelihood of action potentials and temporarily facilitating behaviors related to the subcortical area beneath the target electrode [38]. On the other hand, the cathodal current hyperpolarizes neurons and may inhibit corresponding cortical areas [29,39,40]. tDCS can produce both online and offline effects. Online effects occur during the stimulation, while offline effects or after-effects persist after the stimulation ends [34]. Reported study results support the use of repetitive stimulation at specific time intervals to prolong after-effects and enhance the effectiveness of this technique [40,41]. Multiple sessions conducted on consecutive days create cumulative and sustained neural stimulation, effectively altering the excitability of the brain cortex [38,42]. 

Recent studies on human subjects indicate that the concentration of neurotransmitters in areas affected by tDCS may modulate an individual’s response to stimulation and decision-making processes [32]. Filmer et al. (2017) found that anodal stimulation of the left dorsolateral prefrontal cortex [DLPFC] [43], which is involved in various decision-making and learning processes [26,44,45], along with training, improved performance on a newly-learned decision-making task. Abedanzadeh et al. (2021) demonstrated that the application of transcranial stimulation to the left dorsolateral prefrontal cortex enhanced processing speed and attention capacity, resulting in a reduction in the PRP [37]. However, the findings of Molero-Chamizo et al. (2018) demonstrated that the effect of anodal tDCS applied to the left primary motor cortex (M1) before performing a simple reaction time task is fundamentally dependent on the time interval between stimulation and task execution. Specifically, anodal stimulation immediately before task execution improved motor performance [46]. In a study conducted by Drummond et al. (2017), the researchers aimed to examine the lateral differences in a selective reaction time task. They investigated the effects of concurrent modulation of the left and right motor cortex using dual-hemisphere tDCS and found evidence of increased independent response onset speed, regardless of the limb used, in a forced choice reaction time task [47].

Improving motor performance and the ability to respond quickly are fundamental challenges for the neuro-motor system, which is especially crucial in situations involving fatigue. Research results show that often in the final stages of sports competitions, when both physical and mental fatigue occur, athletes’ technical skills, decision-making abilities, and information processing for performing quick and accurate reactions decrease [1,48,49,50]. These abilities are crucial in combat and competitive sports, where slower reactions than desired reduce the chance of providing an appropriate and timely response to the opponent’s actions, consequently decreasing success in a competition [51].

Mental fatigue is accompanied by a decrease in performance and neural changes in various regions of the brain, particularly the prefrontal cortex and anterior cingulate cortex (ACC) [52,53,54]. The likely mechanism behind the impact of mental fatigue on performance is an elevation in adenosine concentration in the prefrontal cortex. This increase is triggered by cognitive tasks and hampers executive functions [50]. Since in dual-task conditions, the response selection stage is related to executive functions [6,8], interventions to mitigate the detrimental effects of fatigue on performance are essential. Recent research by Nikooharf-Salehi et al. (2021) showed that tDCS applied over the left DLPFC can improve performance impairments in alertness (vigilance) caused by fatigue over time [55]. Anodal stimulation has been found to reduce the effects of fatigue on reaction time [56].

Recently, a few studies have investigated the effect of tDCS on the PRP. Additionally, there have been studies on the impact of tDCS in mitigating the effects of fatigue on cognitive and sports performance. Assuming that PRP can be influenced by fatigue conditions, the researcher wondered if tDCS could also impact PRP when fatigue is present. Additionally, some research results on PRP indicate that the PRP effect decreases when the SOAs between two stimuli are either very short or long [57,58]. Therefore, this study aims to answer the following questions: Can tDCS affect PRP at different SOAs between two stimuli under mental fatigue conditions? Is there a difference between anodal and cathodal tDCS on PRP under mental fatigue conditions?

## 2. Materials and Methods

### 2.1. Participants 

The G*Power software (version 3.1) calculated the sample size of 24 participants with within between subjects ANOVA, a significance value of 0.05, a power of 0.85, and an effect size of 0.3. However, the participants in the present study consisted of 39 male undergraduate students aged 20 to 25 from Shahid Chamran University of Ahvaz. It is worth mentioning that after applying the entrance and exit criteria, 39 out of the initial 45 volunteer students remained in this study. Two people from the first group were excluded due to non-participation in a stimulation session. In the second group, one person was excluded due to illness, and one person was excluded due to the diagnosis of heavy smoking. To make the groups equal, we removed two people from the third group. Finally, 13 participants remained in each group (a total of 39 participants).

Before engaging in any activity in the laboratory, the inclusion criteria of the research participants were checked. Inclusion criteria include right-handedness, normal vision, no history of sedative drug use, no alcohol consumption, no drug addiction, no cardiovascular and respiratory diseases, neurological or mental disorders, and no metal implants. Participants who met all the inclusion criteria were included in this study. Exclusion criteria included any illness or non-compliance with research rules, not participating in a mobility session, withdrawal, or unwillingness to participate in this study.

### 2.2. Study Design

As a study with a quasi-experimental design, the present study was confirmed by the ethics committee of Shahid Chamran University of Ahvaz and recorded (EE/1401.2.24.222390/scu.ac.ir). All the processes involved in this study met the requirements of the Declaration of Helsinki, and the participants completed an informed consent form before taking part in this study. 

### 2.3. Apparatus 

The measurement tools included a consent form for participation in the research project, the Edinburgh Handedness Inventory questionnaire [59], and the Psychological Refractory Period Measuring Apparatus.

**Figure 1:**
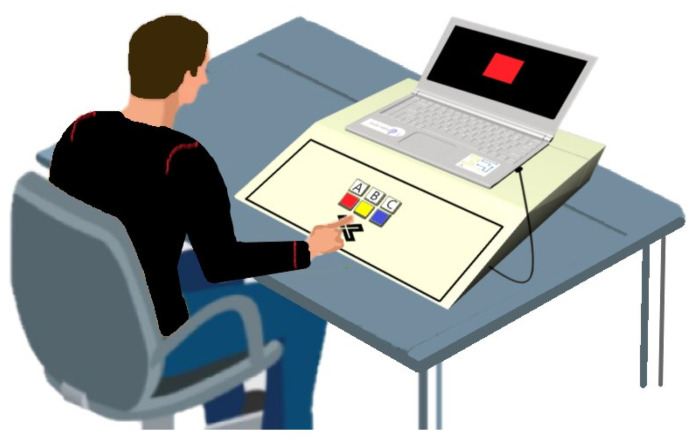
The Psychological Refractory Period Measuring Apparatus.

The measurement tools include the informed consent form for participation in the research project, the Edinburgh-handedness questionnaire (Oldfield, 1971) [59], the transcranial direct current brain stimulation device, model NeuroStim 2, made in Iran, and the Psychological Refractory Period Measuring Apparatus, a product of Medina Teb Gostar Company. This tool is a researcher-made device. the validity of this device has been confirmed by six motor behavior science specialists, and to evaluate the validity of this tool, the simultaneous validity test method was used with the Yagami YB1000 reaction time measurement device. As a result, the correlation coefficient Pearson was 0.81. The reliability of the tool was obtained using the test-retest method for simple reaction time and 0.89, respectively. The Psychological Refractory Period Measuring Apparatus is a device designed by Doustan and his colleagues (2016) in which the interval between presenting two stimuli, the type of stimuli, and the number of stimuli-responses by the tester can be changed [60]. This device has a keypad consisting of a row of buttons with various colors and a row of buttons with capital English letters. It is connected to the la’top’s USB port using a cable. When the participant is in front of the display screen, they should press the button that corresponds to the displayed letter or color as quickly as possible. This allows us to measure their reaction time. The device records the time between the presentation of the stimulus and the partici’ant’s response (Figure 1).

The Stroop task software (version 4658), a product of Sina Company (Tehran, Iran), was used to induce mental fatigue. In this task, 48 congruent and 48 incongruent color words were presented on the screen in blue, red, yellow, and green colors. Congruent words refer to displaying a word in the same color as its meaning, while incongruent words refer to displaying a word in a different color from its meaning. A set of 96 color words was randomly and sequentially displayed. Participants were instructed to disregard the meaning of the words and focus solely on selecting the visual color of the word on the keyboard. To assess the severity of mental fatigue, the Visual Analogue Scale to Evaluate Fatigue Severity (VAS-F) was used [55,61,62]. This scale consists of 18 items related to the experience of mental fatigue. For each item, a visual analog scale was provided, extending between two extreme states (e.g., f“om "not at all t”red"“to "very t”red"). Participants were instructed to mark a single point on the line that represents their current feeling. This questionnaire consists of two subscales: fatigue (items 1–5 and 11–18) and energy (items 6–10). Scores range from 0 to 100, with each line being 100 millimeters long. A score of 0 mm represe“ts "no mental fati”ue," while a score of 100 mm represe“ts "maximum mental fat”gue". The reliability and validity of the initial psychometric evaluations conducted by Lee et al. (1991) showed high internal consistency, ranging from 0.94 to 0.96. Concurrent validity was established with the Stanford Sleepiness Scale and the Mood States Scale [63].

The tDCS device model Neuro-Estim 2, produced by Medina-Teb Gostar Company in Iran, was used for brain stimulation. This device has two completely independent channels that are used to apply stimulations with separate settings. Electrodes with dimensions of 4 × 4 cm (16 cm^2^) were used to determine the electrode placement on the scalp. The international 10–20 system was utilized, which is based on the correlation between the electrode location and the cortical area of the brain. The numbers 10 and 20 refer to the distances between adjacent electrodes, which represent 10% or 20% of the total distance across the skull (front-back or right-left). Additionally, the questionnaire on Adverse Effects (AEs) of tDCS was used to monitor the side effects of tDCS. This questionnaire consists of 14 items, which are rated on a scale from 1 (no sensation) to 10 (severe sensation). It includes potential side effects of tDCS, such as headache, neck pain, scalp pain, tingling, itching, burning sensation, skin redness, sleepiness, difficulty concentrating, and severe mood changes [64]. 

### 2.4. Procedure

The participants arrived at the designated testing location, the Motor Behavior and Sport Psychology Laboratory at Shahid Chamran University in Ahvaz, on the scheduled day, as per a program that had been previously announced to them. All tests and stimulation sessions were conducted between 10:00 am and 1:00 pm and before lunch. The participants were asked to sleep well and not to do any sports or vigorous physical activity before coming to the laboratory. Before engaging in any activity, all participants were asked questions regarding the entry criteria. To determine the dominant hand, all participants were required to complete the Edinburgh Handedness Inventory questionnaire [59]. Those who met the criteria completed a consent form to participate in the research. Subsequently, information was provided about the research process, working with tools, and completing questionnaires. Each participant performed ten experimental trials at ten different SOAs (50, 75, 100, 150, 300, 400, 600, 900, 1200, and 1500 ms), which were presented randomly during the experiment. The SOAs were derived from a previous study on the PRP [10]. However, since we intended to investigate the relationship between SOAs and PRP with a greater number of data points, we increased the number of SOAs compared to previous studies. It should be noted that the participants did not receive any feedback.

**Figure 2:**
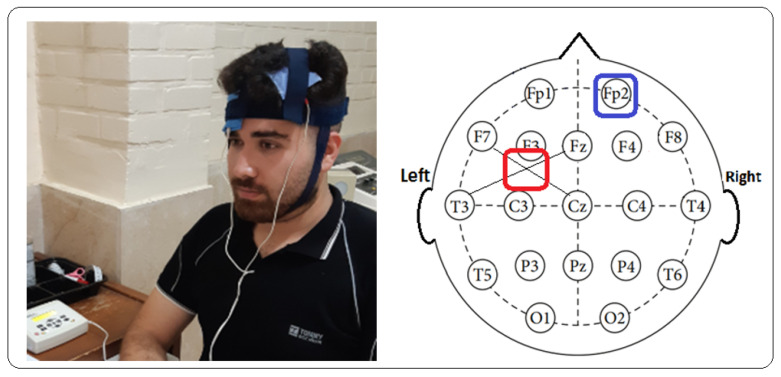
The red square indicates the location of the active electrode, and the blue square indicates the location of the return electrode.

The experiment consisted of seven sessions: a pre-test session, four stimulation sessions, a post-test session, and a follow-up session (refer to Figure 2). All participants were randomly assigned to one of three 13-person stimulation groups: anodal, cathodal, or sham. Each group was divided into two subgroups to control for the effect of the testing order (counterbalancing). One subgroup first took the tests under non-fatigued conditions and then under conditions of mental fatigue, while the other subgroup did the opposite (for more details, refer to the mental fatigue protocol). Participants in the second subgroup took the test first under conditions of mental fatigue. After one hour of rest and leaving the laboratory environment, they took the test again under non-fatigued conditions.

In each trial, participants were instructed to position their right index finger on a specified spot while seated 60 cm away from the monitor. They were also instructed to concentrate their attention on the test. After being instructed to prepare and upon pressing the start button by the experimenter, a beep sound was emitted from the monitor for 100 ms. Following the 2000 ms interval, the initial stimulus, a visual stimulus displaying three reaction time options, appeared on the screen in the form of an English letter (A, B, or C). Participants had to press the corresponding key on the keyboard with their right index finger as quickly as possible. After randomly selecting one of the following SOA (one of the durations: 50, 75, 100, 150, 300, 400, 600, 900, 1200, or 1500 ms), the second stimulus, a visual stimulus, appeared on the screen as a colored square with three reaction time choices: red, yellow, or blue. After the pre-test session, individuals were assigned to the intervention phase, which consisted of two real stimulation groups and one sham stimulation group. In this phase, all participants attended four tDCS sessions on four consecutive days. The anodal group received anodal stimulation, the cathodal group received cathodal stimulation, and the sham group received sham stimulation (for more details, refer to the stimulation protocol). One day after the last stimulation session, all participants took part in the post-test [32,43]. Finally, four days after the post-test session, participants were assessed in the follow-up session. The pre-test, post-test, and follow-up conditions were similar for all three groups, and all procedures, except for the stimulation conditions, remained unchanged (see Figure 2).

Stimulation Protocol: The current density for all types of stimulation was set at 0.0625 mA/cm^2^ (with a current intensity of 1 mA). It was applied for 20 min using a pair of 4 × 4 cm sponge electrodes that were secured onto the specific cortical areas with specialized straps [26,43,65]. Special pads for each electrode were moistened in a 9% saline solution to minimize the apparent resistance [impedance]. They were then placed in the targeted areas [66,67]. Four consecutive stimulation sessions were proposed for each participant [32,36,43]. In the stimulation sessions, the device electrodes in all three groups were connected to the same areas on the scalp using the 10–20 EEG electrode system. The active electrode was placed on the left PLPFC area, specifically 1 cm posterior to F3, which roughly corresponds to the posterior part of Brodmann area 9. The reference electrode was placed on the supraorbital region on the right side, specifically Fp2 [32,36,43]. In the present experiment, there were three stimulation conditions. In the anodal stimulation condition, the active electrode was the anode, and the reference electrode was the cathode. In the cathodal stimulation condition, the cathode served as the active electrode, while the anode served as the reference electrode. For the sham stimulation condition, the electrodes were placed in the same positions as for the real stimulation, but the current was only applied during the first and last 30 s of the stimulation to induce the same skin sensation as in the real stimulation [37,55,62,68].

To ensure blinding, participants were questioned at the end of the sessions about the nature of the experimental session (anodal, cathodal, or sham). Explanations were provided regarding the three groups. Since most of them guessed incorrectly, we concluded that blinding conditions were effective [32].

Side Effects Assessment: To monitor potential side effects, participants were requested to accurately and honestly complete the adverse effects questionnaire suggested by Brunoni et al. (2011) [69] before and after each stimulation session [35]. Although side effects have been reported, including itching under the electrode, burning and redness of the scalp, and mild headaches, both during and after stimulation, they are generally mild and do not have long-term effects [70,71]. Moreover, in clinical trials, no significant side effects have been observed in repeated tDCS sessions with a maximum dose of 2 mA [68,72].

Statistical Methods: In the current study, descriptive statistical methods, such as calculating the mean and standard deviation, were used to analyze the data. The Shapiro–Wilk test was used to check the normality of the data distribution, while Levene’s test was used to assess the equality of variances in the data. Mixed ANOVA methods with repeated measures and one-way ANOVA were used for inferential analysis. Post hoc tests were conducted for pairwise group comparisons using independent t-tests. The significance level for the tests was set at 0.05, and the Bonferroni post hoc test was used to examine the location of the differences. The data analysis was performed using SPSS software version 21. Microsoft Word and Excel version 2016 were used for writing the research report and creating tables and figures.

## 3. Results

This study investigated the effect of tDCS on response time to the second stimulus (RT2) and PRP at different SOAs under both fatigue and non-fatigue conditions. To assess the equality of error variances, Le’ene’s test was employed. Based on the significance levels from all tests, which were higher than 0.05, we concluded that the error variance was equal across all conditions. Therefore, the assumption of using a mixed analysis of variance (ANOVA) was valid. Hence, this test was used, and its results are presented in Table 1.

### 3.1. RT2 Findings

As shown on the right-hand side of Table 1, the results of the composite analysis of variance test for investigating the effect of tDCS on the response time to the second stimulus indicated a significant main effect (F = 58.801, *p* < 0.001, η^2^ = 0.620). This means that regardless of fatigue and SOA conditions, there is a difference in response time to the second stimulus between the pre-test, post-test, and follow-up. The Bonferroni post hoc tests indicated a significant difference between the pre-test and post-test, as well as the follow-up (*p* < 0.001). The mean RT2 in the pre-test, post-test, and follow-up was 914.007, 844.179, and 806.595, respectively. However, the effect of the test on the group (F = 1.284, *p* = 0.288, η^2^ = 0.067), the main effect of fatigue (F = 0.78, *p* = 0.781, η^2^ = 0.002), and the effect of fatigue on the group were not significant (F = 0. 979, *p* = 0.454, η^2^ = 0.052). Nevertheless, the main effect of SOA on the response time to the second stimulus was significant (F = 81.812, *p* = 0.001, η^2^ = 0.694). The Bonferroni post hoc tests showed a significant difference between 50, 75, 100, and 150 SOAs and between 300, 400, 600, 900, 1200, and 1500 SOAs, as well as between 300, 400 SOAs and 600, 900, 1200, and 1500 SOAs (*p* < 0.005). However, the effect of SOA on the group (F = 0. 979, *p* = 0.454, η^2^ = 0.052), the effect of the fatigue test (F = 1. 715, *p* = 0.187, η^2^ = 0.045), and the effect of the test on fatigue in the group (F = 1. 776, *p* = 0.143, η^2^ = 0.090) were not significant.

The effect of the test on SOAs on the response time to the second stimulus was significant (F = 1.798, *p* = 0.057, η^2^ = 0.048). Repeated measures analysis of variance and dependent t-tests were used to find the location of differences. The results showed that regardless of fatigue conditions, the changes in RT2 from the pre-test (F = 44.821, *p* < 0.001, η^2^ = 0.368) to the post-test (F = 480.49, *p* = 0.0001, η^2^ = 0.391) and follow-up (F = 55.222, *p* = 0.0001, η^2^ = 0.418) at different inter-stimulus intervals were significant. This means that there are significant differences in the changes in RT2 from the pre-test to the post-test and then to follow-up at different SOAs.

**Figure 3:**
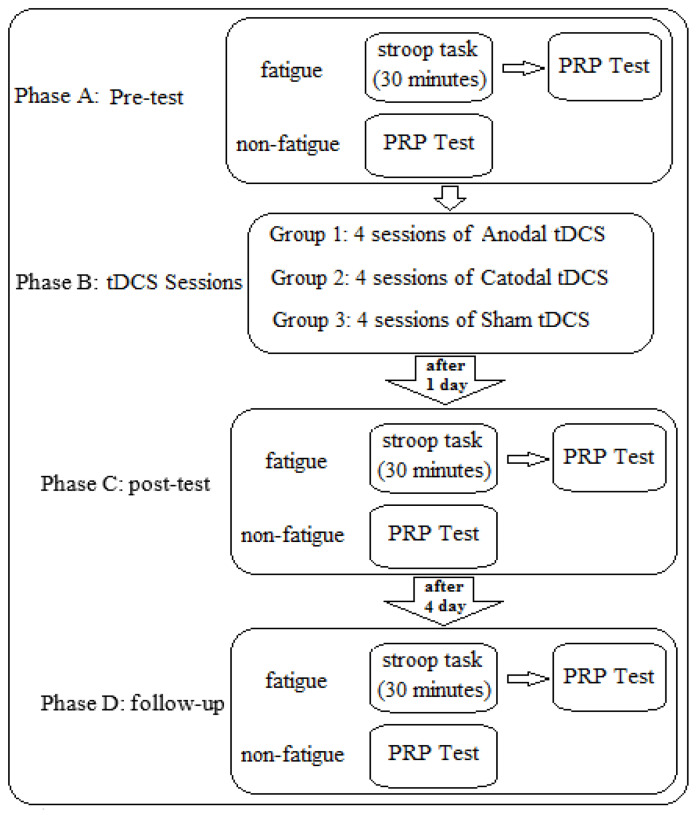
Research implementation protocol.

The results of the Bonferroni post hoc tests showed that in the pre-test, there was a significant difference between SOA 300 and SOAs of 100 and 150 ms, as well as between SOA 400 and SOAs of 50 to 150 ms, and between SOAs of 50 to 400 ms and SOAs of 600, 900, 1200, and 1500 ms (*p* < 0.005). In the post-test, there was a significant difference between SOAs of 50, 75, 100, and 150 ms and SOAs of 300, 400, 600, 900, 1200, and 1500 ms, as well as between SOA 300 and SOAs of 900, and 1200 ms, and between SOA 400 and SOAs of 600, 900, and 1200 ms (*p* < 0.005). In the follow-up, there was a significant difference between SOAs (50, 75, 100, and 150 ms) and SOAs (300, 400, 600, 900, 1200, and 1500 ms), as well as between SOA 400 and SOAs of 900, 1200, and 1500 ms (*p* < 0.005). The findings suggest that regardless of fatigue conditions, the changes in RT2 in each of the ten SOAs were significant. This means that there are significant differences in RT2 between pre-test, post-test, and follow-up at different SOAs (see Figure 3). 

**Figure 4:**
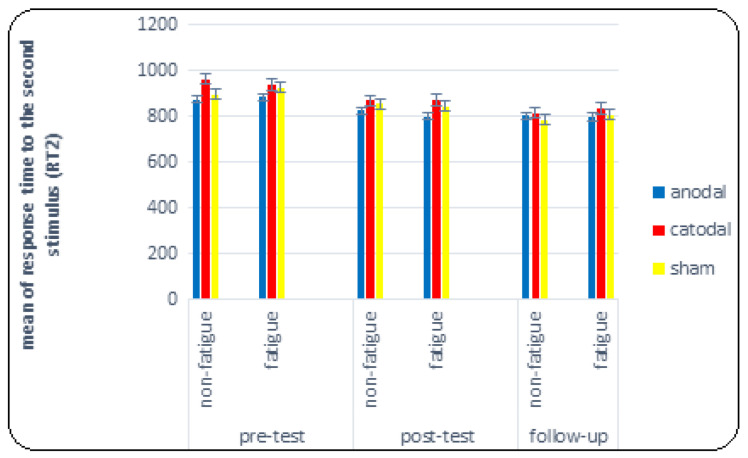
Comparison graph of RT2 (response time to the second stimulus) under fatigue and non-fatigue conditions in pre-test, post-test, and follow-up.

The results of the Bonferroni follow-up tests showed a significant difference between the pretest, post-test, and follow-up (*p* = 0.0001) at a SOA of 50 ms. Also, the comparison of means indicated a reduction in the mean RT2 in the posttest and follow-up compared to the pretest. At SOA of 75 ms, there was a significant difference between the pretest and posttest and follow-up (*p* = 0.0001) and between the post-test and follow-up (*p* = 0.006), and the comparison of means showed a decrease in the mean RT2 in the follow-up compared to the posttest and in the posttest compared to the pretest. At SOA of 100 ms, there was a significant difference between the pretest and posttest and follow-up (*p* = 0.0001) and between the posttest and follow-up (*p* = 0.025), and the comparison of means showed a decrease in the mean RT2 in the follow-up compared to the posttest and in the posttest compared to the pretest. At a SOA of 150 ms, there was a significant difference between the pretest, posttest, and follow-up (*p* = 0.0001), and the comparison of means showed a decrease in the mean RT2 in the follow-up and post-test compared to the pretest. At a SOA of 300 ms, there was a significant difference between the pretest, post-test, and follow-up (*p* = 0.0001) and between the post-test and follow-up (*p* = 0.001), and the comparison of means showed a decrease in the mean RT2 in the follow-up compared to the posttest and in the posttest compared to the pretest (see Figure 4).

At SOA of 400 ms, there was a significant difference between the pretest, post-test, and follow-up (*p* = 0.0001) and between the post-test and follow-up (*p* = 0.001), and the comparison of means showed a decrease in the mean RT2 in the follow-up compared to the posttest and in the posttest compared to the pretest. At SOA of 600 ms, there was a significant difference between pretest and posttest (*p* = 0.048) and follow-up (*p* = 0.0001) and between posttest and follow-up (*p* = 0.015), and the comparison of means showed a decrease in the response time to the second stimulus in the follow-up compared to the posttest and in the posttest compared to the pretest. At SOA of 900 ms, there was a significant difference between the pretest and posttest and follow-up (*p* = 0.0001) and between the posttest and follow-up (*p* = 0.041), and the comparison of means showed a decrease in the response time to the second stimulus in the follow-up compared to the posttest and in the posttest compared to the pretest. 

**Figure 5:**
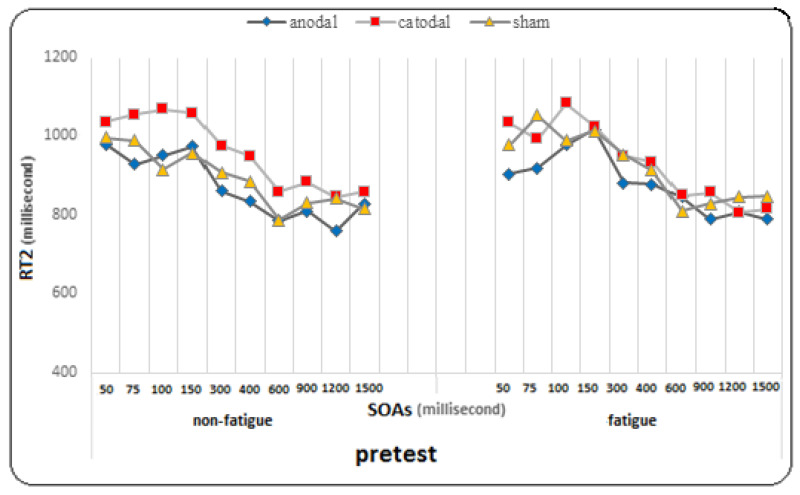
The graph of changes in response time to the second stimulus in different SOAs under fatigue and non-fatigue conditions in the pre-test phase for all three groups.

**Figure 6:**
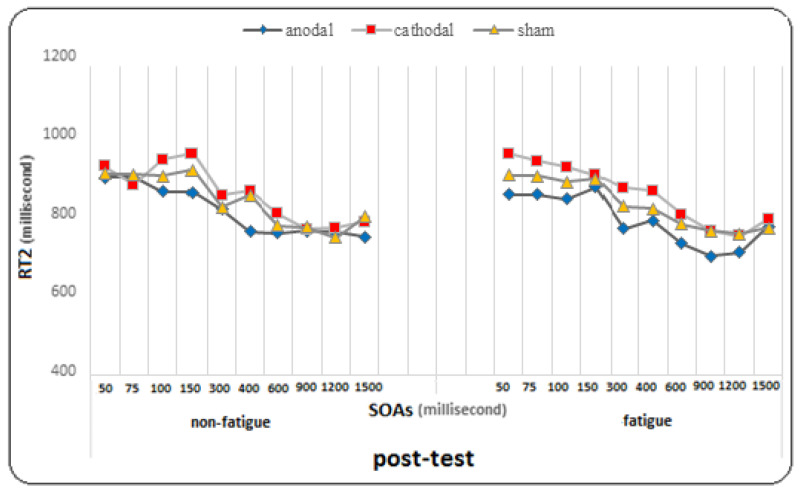
The graph of changes in response time to the second stimulus in different SOAs under fatigue and non-fatigue conditions in the post-test phase for all three groups.

**Figure 7:**
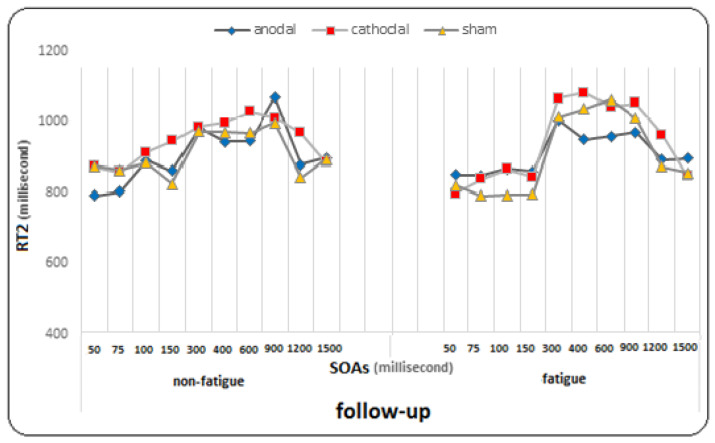
The graph of changes in response time to the second stimulus in different SOAs under fatigue and non-fatigue conditions in the follow-up phase for all three groups.

At SOA of 1200 ms, there was a significant difference between the pretest, post-test, and follow-up (*p* = 0.0001), and the comparison of means showed a decrease in the mean RT2 in the follow-up and post-test compared to the pretest. At SOA of 1500 ms, there was a significant difference between the pretest and posttest and follow-up (*p* = 0.001) and between the posttest and follow-up (*p* = 0.016), and the comparison of means showed a decrease in the response time to the second stimulus in the follow-up compared to the posttest and in the posttest compared to the pretest. However, the effect of the test at SOAs in the group (F = 1.369, *p* = 0.131, η^2^ = 0.071) and the effect of fatigue at SOAs (F = 0.684, *p* = 0.676, η^2^ = 0.019), the effect of fatigue at SOAs in the group (η^2^ = 0.049, *p* = 0.517, F = 0.936), the effect of the test on fatigue at SOAs (F = 1.163, *p* = 0.317, η^2^ = 0.031), and the effect of the test on fatigue at SOAs in the group (F = 0.852, *p* = 0.643, η^2^ = 0.045) did not reach significance for the response time to the second stimulus (For more detailed observations, see the diagrams in Figure 5, Figure 6 and Figure 7).

The study also examined the impact of anodal and cathodal tDCS on PRP at various SOAs, both in fatigue and non-fatigue conditions. The findings are displayed on the left side of Table 1. It is important to mention that PRP was calculated by subtracting the response time to the second stimulus when the first stimulus was present from the response time when it was absent.

### 3.2. PRP Findings

**Figure 8:**
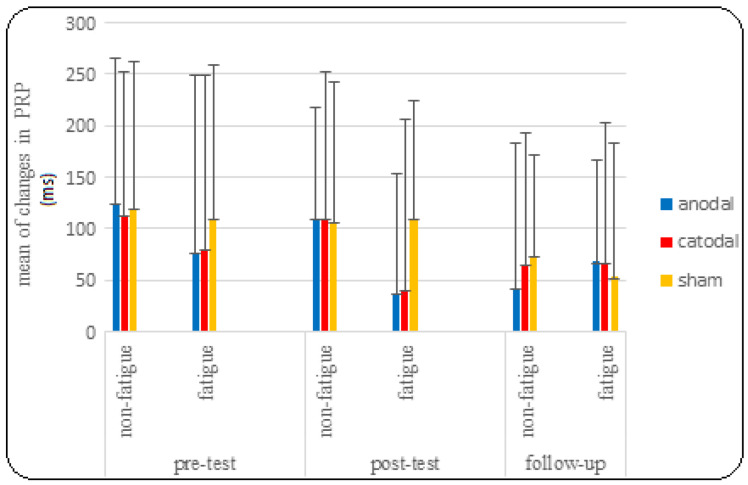
Comparison graph of changes in PRP under fatigue and non-fatigue conditions in pre-test, post-test, and follow-up.

As shown on the left side of Table 1, the results of the composite analysis of variance to investigate the effect of tDCS on PRP showed that the main effect of the test was significant (F = 5.655, *p* = 0.005, η^2^ = 0.136). This means that regardless of fatigue and SOA conditions, there was a significant difference between the PRP variable in the pre-test, post-test, and follow-up. Bonferroni follow-up tests showed a significant difference between post-test and follow-up (*p* = 0.009) in the PRP variable. Additionally, the mean comparison showed that PRP decreased in follow-up compared to post-test and, to some extent, in post-test compared to pre-test. The mean PRP in the pre-test, post-test, and follow-up was 104.281, 85.257, and 61.340, respectively (see Figure 8). 

Nevertheless, the effect of the test did not reach significance in the significant group (F = 0.385, *p* = 0.819, η^2^ = 0.021). However, the main effect of fatigue on PRP was significant (F = 4.908, *p* = 0.033, η^2^ = 0.120). This means that regardless of the test and SOA, the PRP variable showed a significant difference between fatigue and non-fatigue conditions. Bonferroni follow-up tests showed a significant difference between fatigue and non-fatigue conditions in the PRP variable (*p* = 0.033). Additionally, the mean comparison showed that PRP decreased in fatigue conditions compared to non-fatigue conditions. The mean in non-fatigue and fatigue conditions was 96.045 and 71.207, respectively (see Figure 7).

**Figure 9:**
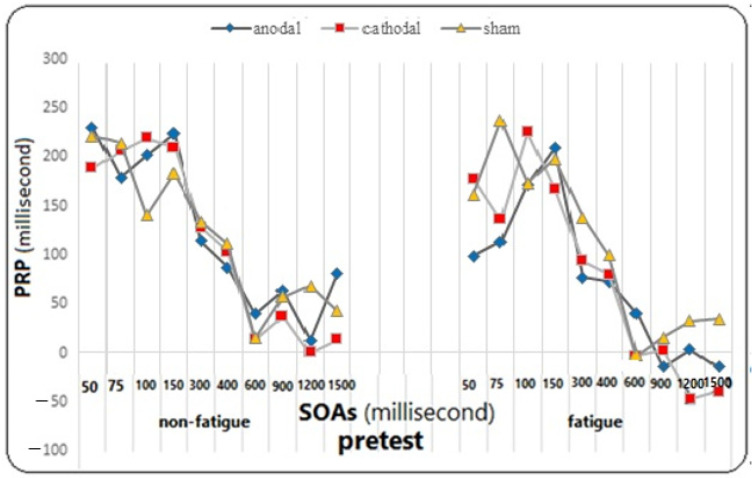
The graph of changes in PRP in different SOAs under fatigue and non-fatigue conditions in the pre-test phase for all three groups.

**Figure 10:**
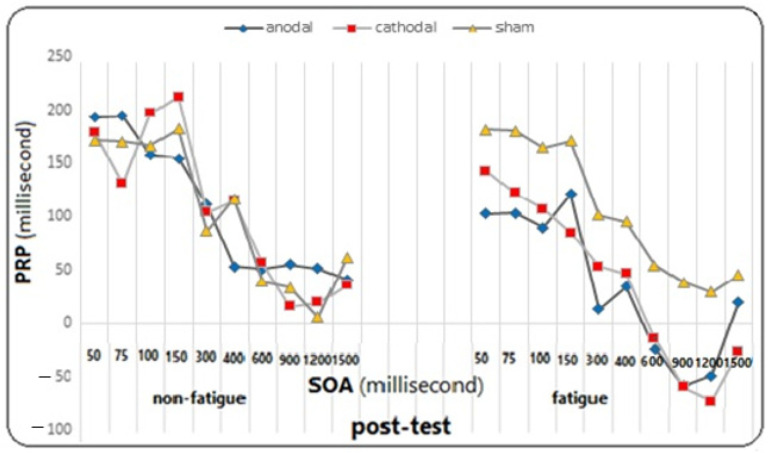
The graph of changes in PRP in different SOAs under fatigue and non-fatigue conditions in the post-test phase for all three groups.

**Figure 11:**
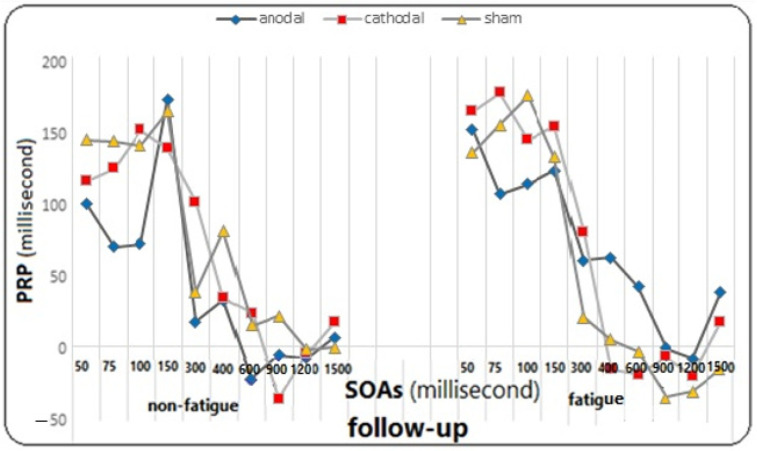
The graph of changes in PRP in different SOAs under fatigue and non-fatigue conditions in the follow-up for all three groups.

On the other hand, the effect of fatigue did not reach significance in the group (F = 0.482, *p* = 0.621, η^2^ = 0.026). However, the main effect of SOA on the PRP variable was significant (F = 81.812, *p* = 0.0001, η^2^ = 0.694), meaning that regardless of the test and fatigue conditions, the mean PRP was different for different SOAs. The Bonferroni follow-up tests showed a significant difference between SOAs of 50, 75, 100, and 150 ms with SOAs of 300, 400, 600, 900, 1200, and 1500 ms, as well as between SOAs of 300 and 400 with SOAs of 600, 900, 1200, and 1500 ms (*p* ≤ 0.005). However, the effect of SOA did not reach significance in the group (F = 0.979, *p* = 0.454, η^2^ = 0.979), and neither did the effect of the fatigue test (F = 1.930, *p* = 0.153, η^2^ = 0.051), the effect of the test in fatigue in the group (F = 1.144, *p* = 0.343, η^2^ = 0.060), the effect of the test in SOAs (F = 1.798, *p* = 0.057, η^2^ = 1.798), the effect of the test in SOAs did not reach significance in the group (F = 1.369, *p* = 0.131, η^2^ = 0.071), the effect of fatigue in SOAs (F = 0.684, *p* = 0.676, η^2^ = 0.019), the effect of fatigue in SOAs in the group (F = 0936, *p* = 0.517, η^2^ = 0.049), the effect of the test in fatigue in SOAs (F = 1.163, *p* = 0.317, η^2^ = 0.031), and the effect of the test in fatigue in SOAs in the group (F = 0.852, *p* = 0.643, η^2^ = 0.045) on the response time to the second stimulus were not significant (for more detailed observations, see the diagrams in Figure 9, Figure 10 and Figure 11).

## 4. Discussion

The present study, which included two stimulation groups and one sham group, investigated the effect of two tDCS methods (anodal and cathodal) on PRP at ten different SOAs under conditions of mental fatigue and non-fatigue. We evaluated both RT2 and PRP. RT2 showed significant improvement during the pre-test, post-test, and follow-up stages. However, there was not a significant difference observed between the groups in this area. There was a difference in response time to the second stimulus at different SOAs. Shorter responses were observed at longer SOAs (600, 900, 1200, and 1500 ms), while longer responses were observed at shorter SOAs (50, 75, 100, and 150 ms). As explained in the PRP effect, the response time to the second stimulus increases with the decrease in the SOA [10]. In the pre-tests, under both conditions of mental fatigue and non-fatigue, the longest response was observed at the SOA of 100 ms, while the shortest responses were recorded at 900 and 1200 ms. This is interesting because a similar finding was reported by Abedanzadeh and Alboghobish (2017), who investigated the effect of Stroop interference and SOAs on PRP [73]. They showed that a longer response time was observed for the non-synchronous-synchronous sequence of stimuli at the SOA of 100 ms, even compared to the 50 ms interval. Additionally, Nouri et al. (2021) conducted a study on the effects of various pre-cue times and SOAs on PRP in athletes [60]. The study revealed that a pre-cue time of 1 second and an SOA of 100 milliseconds led to the longest response time to the second stimulus and the longest PRP.

At SOAs of 900 and 1200 ms, there is an increase in the inter-stimulus interval and possibly the completion of the processing stages of the first stimulus. This reduces the effect of the first stimulus on the response to the second stimulus. As a result, it is not surprising that the response time to the second stimulus is very short at these SOAs. This supports Wolford’s single-channel hypothesis and the results of the study by Pashler and Johnston (1989), which showed that as the SOA increases, the impact of asynchrony between the onset of the two stimuli on the response time to the second stimulus decreases significantly [74].

Interestingly, the conditions changed in the follow-up stage. The shortest response time to the second stimulus was related to shorter SOAs (50, 75, 100, and 150 ms), while the longest response time was related to longer SOAs. Specifically, under fatigue conditions, the longest response time was observed at 300 and 400 ms, while under non-fatigued conditions, it was observed at 600 and 900 ms. This finding is open to interpretation and discussion. The results were almost similar in all three groups. What is clear is that tDCS did not have a significant effect on the response time to the second stimulus. However, what is interesting is that a significant improvement was observed in the follow-up test with shorter SOAs, which was not observed with longer SOAs. Overall, fatigue did not have a significant effect on the response time to the second stimulus. This is consistent with the results of a recent study by Le Mansec et al. (2019), which investigated the effects of mental and physical effort on response time and its components [75]. The study did not observe any negative effect of mental or physical effort on simple response time during voluntary contractions. Many factors may have played a role in performance, such as motivation, which could have partially offset the detrimental impact of fatigue on performance. Additionally, the level of fatigue induced by the task may not have been sufficient to significantly reduce performance [76].

The research results showed that the PRP decreased slightly under conditions of mental fatigue compared to non-fatigue conditions. This finding also confirms the hypothesis that our Stroop task was considered a form of exercise because it is similar to the primary task. Therefore, the delay caused by dual-task situations may have been minimized. By improving performance in the experimental tasks, there was no difference between the groups. It is possible that fatigue had a significant impact, nullifying any actual effect of cathodal tDCS on the PRP. As a result, all groups performed similarly to the sham stimulation conditions. Additionally, the PRP was longer at shorter SOAs of 50, 75, 100, and 150 ms than at longer SOAs, which is consistent with the majority of findings from previous studies on the PRP [18,77]. According to the bottleneck model of response selection, the PRP effect can be explained by the fact that when the time interval between tasks (SOA) is longer, task 1 is fully processed before task 2 reaches the processing bottleneck. Thus, the processing of Task 2 is not delayed by Task 1. However, at shorter SOAs, when the bottleneck processor is still occupied with task 1, task 2 also enters the processing stage. Consequently, the processing of task 2 is delayed until task 1 releases the bottleneck [18]. The PRP showed a significant decrease at SOAs of 900 and 1200 ms. This may have occurred because the processing stages of the initial stimulus response were likely finished within these intervals, allowing the channel to be utilized for the second task. As indicated by the aforementioned results, the shortest responses to the second stimulus were also found within these intervals. In the post-test stage, a significant difference was observed between the fatigue conditions of the groups at an SOA of 1500 ms. Both active stimulation groups performed better than the sham stimulation group in fatigue conditions in the post-test, but no significant difference was observed between the two actual stimulation groups. It should be noted that the difference between the two stimulation groups and the sham group was not observed in the follow-up test. It is clear that the stimulation effects are reduced after 4 days. (Right side of Figure 10, fatigue conditions).

Regarding the effect of anodal stimulation, many studies have shown that anodal stimulation depolarizes neurons, increases the excitability of the cerebral cortex, and improves reaction speed in various tasks [46,56]. Also, Anodal stimulation has been found to reduce the effects of fatigue on reaction time [56]. This type of stimulation temporarily facilitates behaviors related to the subcortical area below the target electrode [38]. Filmer et al. (2017) found that stimulation of the left DLPFC [43], which is involved in various decision-making and learning processes [26,44,45], along with training, improved performance on a newly learned decision-making task.

There is limited information about the actual effects of cathodal tDCS on cognitive performance [78,79,80]. Some researchers have suggested that this may be because the inhibitory effects of cathodal stimulation sometimes cannot sufficiently suppress the target cortical area. On the other hand, the effects of cathodal tDCS can be contradictory. While cathodal stimulation is mostly associated with inhibitory effects on the brain, there is evidence that it can enhance cognition under specific conditions [81]. In a study on adolescents, it was found that 10 min of cathodal tDCS with a direct current of 0.5 mA (using electrodes with a size of 35 square centimeters) significantly reduced the excitability of the brain cortex. However, cathodal stimulation at 1.0 mA resulted in increased excitability [82]. In another study, cathodal stimulation of the motor cortex at an intensity of 1.0 mA demonstrated inhibitory effects, while cathodal stimulation of the same area at an intensity of 2.0 mA resulted in increased excitability [83]. The results of the study by Filmer et al. (2017) showed that cathodal stimulation did not reliably disrupt the training effects compared to the sham stimulation [43]. This indicates that the simplistic “anode-excitation” vs. “cathode-inhibition” model of tDCS effects does not apply to all task conditions. As observed in our study, only minor effects of tDCS were observed in the cathodal group. As there are similarities between the Stroop task and the present research task, our task may be considered a form of exercise. This exercise could potentially help maintain the activity schema and diminish the weak effects of tDCS. On the other hand, the frequency of test repetitions (ten different SOAs in both fatigue and non-fatigue conditions, each with two efforts) may also be considered a form of practice for the task. This practice could have contributed to improved performance and reduced PRP in the post-test and follow-up efforts. As this issue existed in both the active and sham stimulation groups, it can be concluded that this pseudo-exercise may have persisted despite the stimulation, which does not appear to have had a significant effect.

It seems that the general motivational effect of the stimulation, which was present in all three groups, could also be a contributing factor. This is because in all three groups—anodal, cathodal, and sham—the PRP showed improvement in both the post-test and follow-up sessions. Additionally, according to the researcher’s report, the number of response errors was more affected by fatigue than reaction time in conditions of mental fatigue. This issue was observed in all three test sessions. However, since the number of errors was not recorded, it was not possible to make a fair comparison between the groups. Any attempt to mentally compare and express it would be considered biased. Perhaps each of these reasons alone may not reduce or neutralize the effects of anodal and cathodal tDCS, but the cumulative effect of these factors can significantly impact the effects of tDCS on the brain, which has not been firmly established. However, studies aligned with our research have not confirmed the impact of tDCS on motor performance, particularly the speed of response to stimuli. This is evident in studies conducted by Stagg et al. (2011), Horvath et al. (2016), Rabipour et al. (2019), Kimura et al. (2021), and Sevilla-Sanchez et al. (2022) [84,85,86,87,88]. What is striking in the research findings is that a "U-shaped" pattern was observed in all tests, particularly in the shorter SOAs (specifically intervals of 50 and 300 ms). However, there are some irregularities in the pattern due to the variations between the SOAs. In future studies, it is recommended that interstimulus intervals closer to each other, between 10 and 20 ms, be considered in the tests for a more precise and controlled investigation of this issue. 

In our research, PRP was obtained from the second reaction time in the presence of the first reaction time, minus the second reaction time without the presence of the first reaction time. At high SOAs, the presence of the first stimulus-response had a positive effect on the preparation to respond to the second stimulus, while there was no interference with the first response time due to the large distance between the two stimuli. This makes the second reaction time faster in the presence of the first stimulus-response than when the first stimulus-response is not present. Therefore, PRP becomes somewhat negative. This event is quite evident in SOAs of 900 and 1200, especially in mental fatigue conditions. Perhaps the reason that it happened in the mental fatigue condition is that in this situation, the presence of the first stimulus-response creates arousal, which increases the attention of the person and reduces the effect of mental fatigue. In SOAs 1500, this stimulating effect is probably reduced due to the longer time interval between the two stimuli.

Overall, the results showed that the influence of cathodal and anodal tDCS on the response to the second stimulus was minimal. The choice of reaction time, the nature of the task complexity, and the predominance of the effect of test repetition due to the frequency of interstimulus intervals may have minimized the differences between the groups compared to the effect of stimulation. Fatigue did not have a significant effect on PRP, except for low interstimulus intervals. This could be attributed to the similarity between the Stroop task and the main task, as well as the maintenance of the activity schema. Therefore, it is not recommended to use the Stroop task to induce mental fatigue in future studies examining the impact of mental fatigue on reaction time tasks.

## 5. Conclusions

In summary, considering the variation in the impact of different SOAs on response time to the second stimulus, it can be inferred that certain fundamental aspects of the response can be simultaneously processed, and fatigue affects this parallel processing. Therefore, these findings support the phenomenon of summarizing responses in PRP, which, unlike the bottleneck model, predicts an increase in the response time to the first stimulus by increasing the time intervals between the presentation of two stimuli. Furthermore, since in lower SOAs, the response time to the second stimulus was shorter and the PRP was longer, it appears that the execution phase of the first stimulus and the identification phase of the second stimulus are processed in parallel. This finding contradicts the bottleneck model and confirms the hypothesis of capacity sharing. In addition, the effects of cathodal and anodal tDCS on both responses to the first and second stimuli were very small. Therefore, it is suggested to do more research in this field for more clarification.

## Figures and Tables

**Table 1:** Results of the mixed analysis of variance (3 × 3 × 2 × 10) to examine the difference between RT2 and PRP in various tests at different SOAs under fatigue conditions in three groups.

	PRP			RT2		
Source	Partial Eta Squared	Sig	F	Partial Eta Squared	Sig	F
TEST	0.136	0.005 *	5.655	0.620	0.0001 *	58.801
TEST * group	0.021	0.819	0.385	0.067	0.288	1.284
FATIGUE	0.120	0.033 *	4.908	0.002	0.781	0.78
FATIGUE * group	0.026	0.621	0.482	0.043	0.457	0.801
SOA	0.694	0.0001 *	81.812	0.694	0.0001 *	81.812
SOA * group	0.052	0.454	0.979	0.052	0.454	0.979
TEST * FATIGUE	0.051	0.153	1.930	0.045	0.187	1.715
TEST * FATIGUE * group	0.060	0.343	1.144	0.090	0.143	1.776
TEST * SOA	0.048	0.057	1.798	0.048	0.057 *	1.798
TEST * SOA * group	0.071	0.131	1.369	0.071	0.131	1.396
FATIGUE * SOA	0.019	0.676	0.684	0.019	0.676	0.684
FATIGUE * SOA * group	0.049	0.517	0.936	0.049	0.517	0.936
TEST * FATIGUE * SOA	0.031	0.317	1.163	0.031	0.317	1.163
TEST * FATIGUE * SOA * group	0.045	0.643	0.852	0.045	0.643	0.852

* in the first column “Source” indicates the interaction effect; * after the number indicates that *p* < 0.05 is significant.

## Data Availability

The data presented in this study are available on request from the corresponding author. The data are not publicly available due to privacy.
